# Economic Assessment of Waterborne Outbreak of Cryptosporidiosis

**DOI:** 10.3201/eid2310.152037

**Published:** 2017-10

**Authors:** Aksana Chyzheuskaya, Martin Cormican, Raghavendra Srivinas, Diarmuid O’Donovan, Martina Prendergast, Cathal O’Donoghue, Dearbháile Morris

**Affiliations:** National University of Ireland Galway, Galway, Ireland (A. Chyzheuskaya, M. Cormican, R. Srivinas, D. O’Donovan, M. Prendergast, D. Morris);; Health Service Executive, Galway (M. Cormican, D. O’Donovan);; Teagasc, Athenry, Ireland (C. O’Donoghue)

**Keywords:** economic assessment, costs, Cryptosporidium, Cryptosporidium hominis, protozoa, parasite, cryptosporidiosis, infection, water, waterborne outbreak, Ireland

## Abstract

In 2007, a waterborne outbreak of *Cryptosporidium hominis* infection occurred in western Ireland, resulting in 242 laboratory-confirmed cases and an uncertain number of unconfirmed cases. A boil water notice was in place for 158 days that affected 120,432 persons residing in the area, businesses, visitors, and commuters. This outbreak represented the largest outbreak of cryptosporidiosis in Ireland. The purpose of this study was to evaluate the cost of this outbreak. We adopted a societal perspective in estimating costs associated with the outbreak. Economic cost estimated was based on totaling direct and indirect costs incurred by public and private agencies. The cost of the outbreak was estimated based on 2007 figures. We estimate that the cost of the outbreak was >€19 million (≈€120,000/day of the outbreak). The US dollar equivalent based on today’s exchange rates would be $22.44 million (≈$142,000/day of the outbreak). This study highlights the economic need for a safe drinking water supply.

*Cryptosporidium* spp. are protozoan parasites that might be present in inadequately treated water. Human infection can result in watery diarrhea, stomach cramps, bloating, vomiting, and fever (*1*–*3*). Although usually a self-limiting illness in otherwise healthy persons, cryptosporidiosis might be associated with chronic gastrointestinal sequelae in some persons and might be fatal for persons with impaired immune function (*4*). In Ireland, animal contact is the main source of transmission (*5*). Endemic disease usually occurs in the spring, is predominantly rural, and is generally associated with *C. parvum* (*4*,*6*). *C. hominis* is primarily a parasite of primates, including humans, and is less common in Ireland.

Several large waterborne outbreaks of cryptosporidiosis have been reported (*5*,*7*,*8*). In 1993, contamination of the municipal water supply affected an estimated 403,000 persons in Milwaukee, Wisconsin, USA (*7*). In March 2001 in North Battleford, Saskatchewan, Canada, an estimated 7,000 persons became ill from contaminated water (*9*).

Many studies have estimated the economic costs of microbial contamination of drinking water supplies (*7*,*10*–*13*). However, there is no standard method for performing such analysis. Halonen et al. estimated the cost of the lost workdays to be €1.8–2.1 million (*11*). Corso et al. included medical costs and loss of productivity related to cryptosporidiosis in Milwaukee and estimated the costs to be $96.2 million (*7*). A similar approach was used in a study in Canada by the Safe Drinking Water Foundation (Saskatoon, Saskatchewan, Canada) in 2015, whichconducted a full cost-benefit analysis of microbial contamination of the water supply in Walkerton, Ontario (*13*). Regardless of the approach used by researchers, there is agreement that costs of outbreaks are considerable and that benefits of preventive measures need to be investigated (*11*–*13*).

Outbreaks of cryptosporidiosis are common in Ireland. During 2011–2014, a total of 100 outbreaks (84 of which were outbreaks in families) were reported that included 305 cases (*5*). In March 2007, the largest outbreak of cryptosporidiosis in Ireland since surveillance began was identified and was associated with contamination of the public water supply serving an urban area (Galway, Ireland) and surrounding areas. The outbreak was distinguished from the usual spring peak not only by the number of cases but also by the predominantly urban distribution and the infecting species (*C. hominis*). The outbreak lasted for 5 months, by which time there were 242 confirmed cases of cryptosporidiosis (Health Service Executive [HSE], Galway, Ireland, unpub. data), although it was likely that the actual number affected was higher (*14*). A boil water notice was put in place for the duration of the outbreak (158 days); the order affected ≈120,432 persons. The outbreak ended in August 2007 after major investments by local authorities in water treatment infrastructure and major disruption to residents and local businesses.

Water quality incidents, such as the outbreak of cryptosporidiosis in Galway in 2007, can have major economic effects on the entire community (residential and commercial) (*7*,*15*). The challenge associated with managing waterborne infection with *Cryptosporidium* spp. is that infection is not inactivated by chlorination of water, which is the primary method of water treatment in many areas (*6*). Several technologies are available to remove or inactivate *Cryptosporidium* spp. during water treatment. These technologies include filtration and ultraviolet light treatment systems.

We assessed economic costs associated with the waterborne outbreak of cryptosporidiosis in Galway, Ireland, during 2007 from the societal perspective. Assessment of costs included not only costs incurred by the 242 reported cases but costs incurred by persons who were ill but did not seek healthcare, as well as by the wider public, local businesses, the healthcare system, local authorities, national agencies, and tourism. We also examined the ratio between the investment needed to mitigate risk for contamination with *Cryptosporidium* spp. and costs averted by such an investment. The aim of such analysis was to aid decision makers with public investment decisions and to inform other stakeholders about economic consequences associated with outbreaks of this type.

## Materials and Methods

### Costs

A societal perspective was adopted in estimating costs associated with this outbreak. The costs associated with the waterborne outbreak of cryptosporidiosis in Galway in 2007 have a multilevel structure (Figure). Consistent with previous research and traditional health economic frameworks, we included direct and indirect costs in calculations (*15*). Direct costs were medical and healthcare costs, cost of provision of alternative water, and response costs. Indirect costs were loss of income, loss of business, and loss of productivity (*15*). We describe the economic effect of the outbreak on those directly affected, and in the wider community, local businesses, and government agencies. The economic cost estimated in our analysis is based on totaling direct and indirect costs incurred by public and private economic agencies (Figure) associated with the outbreak.

**Figure:**
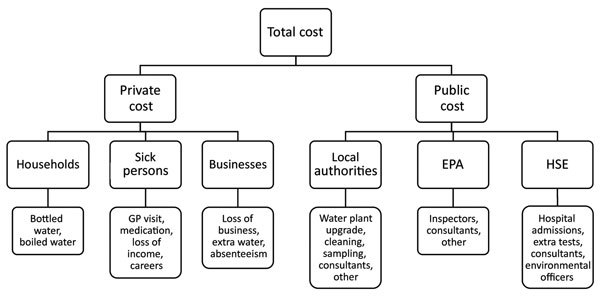
Multilevel structure of costs associated with waterborne outbreak of cryptosporidiosis, Galway, Ireland, 2007. EPA, Environmental Protection Agency of Ireland; GP, general practitioner; HSE, Health Service Executive.

Costs included are consistent with those included in previous economic assessments of waterborne outbreaks of infectious diseases (*7*,*15*,*16*). In our analysis, most costs are reported to have occurred during the outbreak in 2007. Thus, use of a discount rate was not considered necessary. Although the final update was completed in 2009, we could not differentiate what proportion of this update occurred in what period. Thus, the value for the update that was provided to us was not discounted.

### Data Sources and Assumptions

Data, sources, and assumptions (if any) made in estimating costs are provided (Table 1). To estimate household costs, we used data for the Simulation Model of the Irish Local Economy (SMILE) (*17*) to determine the number of households and their socioeconomic characteristics if located in the boil water notice area. SMILE (*17*) is a synthetic dataset that is spatially representative of households and farms at an electoral district level and includes several datasets (e.g., the Living in Ireland Survey, Small Population Statistics, and the Geo-Directory) (*16*).

**Table 1:** Description of costs included in economic assessment of waterborne outbreak of cryptosporidiosis, Galway, Ireland, 2007*

Characteristic	Value and assumption	Source
Private sector		
No. households	45,160	(*17*)
Cost of extra bottled water	48% reported buying bottled water; 80% increased bottled water use from 3.2 L to 16 L/wk; 20% increased use from 3.2 L to 20 L/wk; €0.50/L	HSE, 2007; estimated bottled water retail price
Cost of boiling water	Use of boiled water/household: drinking >12%, 2.1L/adult/d, 1 L/child/d; cooking >30%, 2 L/household/d; dishwashing >43%, 10 L/household/d; hygiene >14% 250 mL/person/d; €0.01/L	(*15*)
Sick persons		
No. reported	242	HSE, 2007
No. not reported	498, 71% not reported	(*16*); HSE, 2007
GP visits	€50/visit, 1 GP visit/confirmed case; all reported patients consulted GP; GMS cost €50.46 (assumed to be €50)	HSE primary care reimbursement service, 2008
Self-medication	€9.26 for antidiarrheal medication; €6.99 for ORS (1 pack); 30% self-medication for reported; 17.6% self-medication for not reported	Pharmacy prices; (*7*)
Loss of income	€122.85/d; 5 d of work missed (all nonhospitalized reported patients and 17.4% of not reported patients); 10 d of work missed for hospitalized patients	Central Statistics Office, 2007; HSE, 2007
Loss of income for caregivers	€122.85/day; children <15 and persons >65 years of age (n = 195); 1 caregiver took 5 d off from work; average 10 d off from work for hospitalized patients	Central Statistics Office, 2007; (*4*); HSE, 2007
Cost of missing college/school	Reported: €69.20/student/d; 5 d missed (10 d for hospitalized patients); not reported: 19% took time off from school/college; average 5 d missed, €69.20/student/d	Central Statistics Office, 2007; (*16*); HSE, 2007
Cost to businesses		
Productivity loss	€134/person/d; average 5 d; 10 d for hospitalized persons and caregivers	Health and Safety Times, 2011
Hotels, B and Bs, hostels		
Extra water	4.2 L/room/d; 2.1 L/person/day	(*18*)
Cancellations	13% cancelation rate; 57% occupancy rate	IPSOS Mori survey
Other assumptions	No. businesses: 70 hotels, 134 B and B, and 18 hostels; no. rooms: 79/hotel, 5/B and B, and 134/hostel; price: €66 hotels, €65 B and B, and €17 hostels	
Care industry†		
Bottled water	2.1 L/person/d	(*18*)
Boiled water	1.5 L/person/d	
Other factors	18 nursing homes in boil water–alert area; average no. residents 41, total no. residents for analysis 742; 168 nurseries/day care centers and 19 child minders; assume 3 children/child minder; average 29 children/nursey or day care center; no. children in nurseries or day care centers 4,929	
Public sector costs, €		
Galway City Council	3,388,840.33	Galway City Council
Galway County Council	2,472,837	Galway County Council
EPA	20,000	EPA
HSE		
Emergency department cost	100; 1.3% of reported patients visited ED	HSE, (*14*)
5,810 extra species detection tests	46.06	University Hospital Galway (no. laboratory tests); commercial service provider (cost)
3,000 extra fecal culture tests	59.58	University Hospital Galway (no. laboratory tests); commercial service provider (cost)
Hospital admissions	753/person/d; 35% admitted to hospital for 10 d	HSE, 2007
Response team (representatives from all public sector categories)		
Opportunity cost of labor	356/consultant/meeting; 16 persons; 28 meetings	IPS

*B and Bs, bed and breakfast; ED, emergency department; EPA, Environmental Protection Agency of Ireland; GMS, general medical scheme; GP, general practitioner; HSE, Health Service Executive; IPS, Institute of Public Administration; ORS, oral rehydration solution. †Nursing homes, nurseries, and day care centers.

In the context of this analysis, we used SMILE data for 2006. On the basis of these data, there were 45,160 households in the area containing 120,432 persons, which included 5,034 children <5 years of age, 13,471 children >5–<18 years of age, 87,970 adults <65 years of age, and 13 persons >65 years of age.

### Not Reported Case-Patients

Many studies have reported variation in the number of not reported cases of gastroenteritis in outbreak settings (*7*,*14**–**15*). Fitzgerald et al. reported that 71% of persons with gastroenteritis whose health was affected were not reported as case-patients (*14*). Corso et al. estimated that during the outbreak of cryptosporidiosis in Milwaukee in 1993, a total of 25% of the population in the area were affected, but 88% of them were not reported (*7*). If the estimate of 25% of the population is applied in this study, the estimated number of cases not reported would have been 25,291.

For our economic assessment, we adopted a more conservative approach and assumed that 71% of persons with signs or symptoms were not reported as case-patients. In addition, we assumed that cases not reported would be in less vulnerable persons 5–64 years of age (≈101,441 persons or 84% of total population in the area estimated by using SMILE data). Therefore, on the basis of the number of reported cases, we estimated that 498 persons who were ill did not seek healthcare or were not reported. The costs we estimated are based on the most conservative figure, but we acknowledge that there is substantial uncertainty regarding the number of persons infected, and the actual number infected may be >498 persons.

We assumed that all confirmed case-patients visited a general practitioner at least once (estimated cost/visit €50). The same cost was assigned to all private patients, public patients, or general medical services patients because general medical services general practitioners claim for out-of-house services is €50.64 (HSE primary care reimbursement service data for 2008). We assumed that 30% of reported case-patients and 17.6% of not reported case-patients self-medicated (*7*) with an antidiarrheal agent and an oral rehydration solution. We also assumed that each self-medicated case-patient purchased 1 packet of antidiarrheal medication and 1 packet of oral rehydration solution (Table 1).

Shortly after the outbreak, the HSE Western Area (Limerick, Ireland) commissioned Ipsos MORI (London, UK), a private marketing research company, to conduct a postoutbreak survey to determine the effect of the outbreak on residents of the area affected by the boil water notice and for persons visiting the area for work or recreational activities (commuters and tourists). Results of this survey were available, and we used these results in our economic assessment. The survey found that all nonhospitalized reported cases-patients and 17.4% of not reported case-patients missed on average 5 days of work, and those who were hospitalized (35% of reported case-patients) were absent from work for an average of 10 days. Because of uncertainty about employment status and sector of persons under consideration, we assumed that all persons 22–65 years of age were employed, received an average industrial wage, and were not paid for days of work missed because of illness (HSE, 2007, unpub. data).

We assumed that dependents (189 symptomatic children <15 years of age and 6 elderly persons >65 years of age) would require a full-time caregiver for the duration of their illness (5 days for nonhospitalized case-patients and 10 days for hospitalized cases-patients). Loss of income (for reported and not reported case patients) was estimated as the average industrial wage rate in Ireland in 2007 (€122.85/d for 2007; Central Statistics Office, Cork, Ireland). Moreover, persons <21 years of age (reported and not reported case-patients) were assumed to either attend college/school or were unemployed. Consistent with previous research by Safefood (*16*), we assigned the opportunity cost to the time they were ill at the minimum wage rate in 2007 (≈€69.20/d for 2007; Central Statistics Office).

On the basis of the Ipsos MORI survey results, we assumed that 48% of households bought extra bottled water, 80% increased bottled water consumption from 3.2 L/wk to 16.1 L/wk, and the remaining 20% increased consumption from 3.2 L/wk to 20 L/wk, at a cost of €0.50/L of bottled water purchased. On the basis of the study by Ailes et al. (*15*), we assumed that 12% of households boiled water for drinking, 30% for cooking, 43% for dishwashing, and 14% for hygiene use, at a cost of €0.01/L. This cost was based on National Electricity Supply Board (Dublin, Ireland) tariffs reported in 2007 and energy required. Further assumptions about the number of liters boiled for each purpose are provided (Table 1).

### Public Sector Costs

We made assumptions related to costs incurred by the public sector. These assumptions include cost of emergency department attendance, which is assumed to apply to 1.3% of reported case-patients (*14*) (€100/visit, which is usually charged to private patients in Ireland (HSE data); cost of a hospital stay (€753/d reported by the HSE in 2015); costs incurred by local authorities (Galway City Council €3,388,840.33 and Galway County Council €2,472,837); cost of the response team (28 meetings attended by 16 senior representatives of HSE West, Galway County Council, and Galway City Council representing an estimated opportunity cost of €356/person/meeting); and costs incurred by the Environmental Protection Agency (Dublin, Ireland), which were estimated to be €20,000.

### Private Sector Costs

Costs to the private business sector in the area proved to be more challenging to estimate because of lack of available data. It proved particularly difficult to obtain reliable data on costs incurred by restaurants. Therefore, we excluded these costs from calculations. The remaining business costs (service sector [lodging businesses] and care sector [nurseries, child care, nursing homes]), sources, and assumptions are provided (Table 1).

## Results

We estimated that the overall cost of the waterborne outbreak of cryptosporidiosis in Galway in 2007 was €19 million or €120,000/day of the outbreak (Table 2). The US dollar equivalent based on today’s exchange rates would be $22.44 million (≈$142,000/day of the outbreak). The estimated cost to households in the affected area was ≈€3.9 million. This estimate translates into an average cost of alternative water of ≈€87/household in the boil water notice zone (or €0.55/household/day of the outbreak). The overall loss of income to households with symptomatic persons was estimated to be €287,957. This cost includes the loss of income of ill persons and their caregivers. The cost for not reported case-patients was estimated to be €74,002. If the average household income in the boil water zone was estimated to be €27,251/year (as estimated by using SMILE data) or €11,796 in 158 days, the loss translates into 0.8% of household income in the affected area during the outbreak.

**Table 2:** Overall estimated cost of waterborne outbreak of cryptosporidiosis, Galway, Ireland, 2007*

Category	Cost, €
Private sector costs	
Household costs	
Bottled water	3,552,299
Boiled water	400, 162
Sick (reported and not reported) costs	
Sick reported	300,236
Sick reported wage loss	36,339
School days lost	89,074
General practitioner	12,100
Self-medication (reported)	1,180
Caregiver income loss (reported)	161,544
Sick not reported	74,002
Sick not reported wage loss	52,973
School days lost (not reported)	1,922
Self-medication (not reported)	1,418
Caregiver income loss (not reported)	17,689
Business costs	
Hotel cancelations	5,374,115
Hotel bottled water bill	1,734,285
Nurseries, day care centers, and nursing homes water bills	525,929
Caregiver productivity loss	
Sick reported	176,206
Sick not reported	19,294
Sick productivity loss	
Reported	36,554
Not reported	57,781
Public sector costs	
Local authorities	
Galway City Council	3,388,840
Galway County Council	2,472,837
EPA	20,000
HSE	
Accident and emergency	315
Hospital	637,791
Extra laboratory tests	446,349
Response team†	159,488
Total cost of outbreak	19,750,722

*EPA, Environmental Protection Agency of Ireland; HSE, Health Service Executive. †Local authorities, EPA, and HSE.

We estimated that cost for lodging and care businesses was €8 million (€50,000 lost by local businesses/day of the outbreak). We also estimated that businesses in the lodging sector lost €5.4 million because of cancellations and an additional €1.7 million required for provision of alternative water to customers. Care businesses provided alternative water to persons in their care at an estimated cost of €525,929.

## Discussion

The waterborne outbreak of cryptosporidiosis that occurred in Galway in 2007 resulted in 242 reported cases of illness and a conservative estimate of 498 additional cases that were not reported. This outbreak also generated a considerable cost to residents, visitors, public bodies, and local businesses. This study highlights the economic need for a safe drinking water supply by reporting public expenditure on mitigating results of the outbreak and private costs to households and businesses in the area. The outbreak was believed to have occurred because the lake that serves as the source of drinking water for the city became contaminated with *C. hominis*, and the treatment process in place was not sufficient to eliminate or inactivate the parasite before water was distributed in the municipal supply.

Our results indicated that there are economic benefits of investing in safe drinking water supplies and water treatment enhancement (e.g., treatment with UV light, which effectively inactivates *Cryptosporidium* oocysts) (*19*,*20*). Hutton et al. reported a return of $5–$46 per $1 investment in water and sanitation improvements: all water improvement interventions examined in their study were cost-beneficial (*12*).

We recognize a major limitation in our approach to assessing relative costs. There is uncertainty associated with number of reported and not reported cases. The outbreak was the largest reported in Ireland, but other studies support the conclusion that when public water supply is contaminated with *C. hominis*, large proportions of populations are affected (*7*,*21*). There is also no basis on which to estimate the frequency with which a source water contamination event likely to result in a comparable outbreak occurs. If such a contamination event occurs frequently (e.g., annually), the cost of implementation greatly outweighs the associated costs of infection. If such an event occurs every 100 years, then the situation might be reversed. In the context of a municipal supply based on a large surface water body in which source protection is challenging, we believe contamination is likely to occur relatively frequently. Methods to define the annual probability of a major contamination event for a particular water supply more precisely would be of value.

Costs assessed for this evaluation related to the period of an outbreak. However, there is reason to believe that some economic impacts continued for years afterward related to the undermining of public trust in the water supply and affected Galway area and local businesses because of reduced numbers of visitors. As many as 13% of respondents to the Ipsos MORI survey indicated that they were less likely to return to the Galway area because of the outbreak. Thus, the economic effect might be greater.

Limited data is one of the obstacles that resulted in the number of assumptions made in this study. Thus, our results should be interpreted carefully, and we advise careful examination of assumptions before drawing conclusions. The lack of data related to businesses in the area and the effect of the outbreak on business operations prevented us from accounting for these effects in our calculations. Moreover, there is an uncertainty about the number of persons who were ill as a result of the outbreak, but did not seek help.

We estimated that the overall cost of this outbreak was €19 million (€120,000/day of the outbreak). These findings strongly support the value of a sustainable economic model to ensure that water infrastructure upgrades anticipate and prevent outbreaks. This study identified that availability of appropriate data are a limiting factor in completion of such economic assessments and provided valuable evidence that investment in safe drinking water supplies and water treatment enhancement benefits public health and the wider economy.
